# A Little Poet

**Published:** 1889-05

**Authors:** 


### A LITTLE POET.

Out in the garden, wee Elsie
   Was gathering flowers for me ;
"O, mamma," she cried, "hurry, hurry,
   Here's something I want you to see."

I went to the window. Before her
   A velvet-winged butterfly flew,
And the pansies themselves were not brighter
   Than the beautiful creature in hue.

"O, isn't it pretty ?" cried Elsie,
   With eager and wondering eyes,
As she watched it soar lazily upward
   Against the soft blue of the skies.

"I know what it is, don't you, mamma ?"
   O, the wisdom of these little things
When the soul of a poet is in them,
   "It's a Pansy—a Pansy with wings."

—*Vicks' Magazine.*

